# Diseases Admitted as in and Out-Patients of the Physicians of the Westminster Hospital, between the 20th of May and the 20th of June

**Published:** 1799-07

**Authors:** 


					Dijeafes admitted as In and Out-Patients of the Phyficians of the Wejl-
m'mfler Hofpltal, between the loth of May and the loth of June.
No. of Cases.
Fever - - 13
Ague 2
Amenorrhcea 9
Anafarca - - 2
Afcites - - - 2
Afthenia - - 4
Afthma - 2
Catarrh - - 2
Colic - - 1
Cough - - 20
Cephaloea - - 2
Diarrhcea - - 4
Dyfpepfia - -3
Enterodynia - 3
Eryfipelas 4
Ha^moptoe - - 1
Haemorrhois - 2
Hypochondriacs - 1
No. of Cases.
p] yfteria i
Hydrocephalus - - 1
Impetigo 2
Itch 7
Lepra Grsecorum - 1
Leucorrhoea - - 4
Menorrhagia - - 3
Phthifis ... I
Paralyfis - - 2
Pleurify 4
Podagra - - l
Rheumatifm - - 11
Struma - 2
Tinea 2
Urticaria 3
4Vertigo - 2
Vomiting - 2
Worms 3
Fevers and pulmonic afFettions have become more frequent in this part of
the town, during the laft month, than in any preceding part of the fpring?
The fever prevalent at prefent differs considerably from the typhus of the
cold months j and appears already to be modified by the ftate of the bile.
t.b.
IjJl

				

